# Depression is associated with reduced outcome sensitivity in a dual valence, magnitude learning task

**DOI:** 10.1017/S0033291723002520

**Published:** 2024-02

**Authors:** Erdem Pulcu, Wanjun Lin, Sungwon Han, Michael Browning

**Affiliations:** 1 Department of Psychiatry, University of Oxford, Oxford, UK; 2 University College London, Max Planck University College London Centre for Computational Psychiatry and Ageing Research, London, UK; 3 Nuffield Department of Medicine, University of Oxford, Oxford, UK; 4 Oxford Health NHS Foundation Trust, Oxford, UK

**Keywords:** Affective bias, computational modeling, depression, reinforcement learning, reward sensitivity

## Abstract

**Background:**

Learning from rewarded and punished choices is perturbed in depressed patients, suggesting that abnormal reinforcement learning may be a cognitive mechanism of the illness. However, previous studies have disagreed about whether this behavior is produced by alterations in the rate of learning or sensitivity to experienced outcomes. This previous work has generally assessed learning in response to binary outcomes of one valence, rather than to both rewarding and punishing continuous outcomes.

**Methods:**

A novel drifting reward and punishment magnitude reinforcement-learning task was administered to patients with current (*n* = 40) and remitted depression (*n* = 39), and healthy volunteers (*n* = 40) to capture potential differences in learning behavior. Standard questionnaires were administered to measure self-reported depressive symptom severity, trait and state anxiety and level of anhedonic symptoms.

**Results:**

Our findings demonstrate that patients with current depression adjust their learning behaviors to a lesser degree in response to trial-by-trial variations in reward and loss magnitudes than the other groups. Computational modeling revealed that this behavioral signature of current depressive state is better accounted for by reduced reward and punishment sensitivity (all *p* < 0.031), rather than a change in learning rate (*p* = 0.708). However, between-group differences were not related to self-reported symptom severity or comorbid anxiety disorders in the current depression group.

**Conclusion:**

These findings suggest that current depression is associated with reduced outcome sensitivity rather than altered learning rate. Previous findings reported in this domain mainly from binary learning tasks seem to generalize to learning from continuous outcomes.

## Introduction

Major depressive disorder (MDD) is a psychiatric condition associated with symptoms such as low mood and lack of interest in things previously deemed pleasurable (i.e. anhedonia) (American Psychiatric Association, 2013). Existing evidence suggests that MDD is also associated with impairments in cognitive domains such as decision-making (Pulcu, Thomas et al., 2014; Pulcu, Trotter et al., 2014). The reinforcement learning (RL) framework has been instrumental in identifying different sources of decision-making impairments observed in patients with MDD, as RL tasks simultaneously probe different dimensions of human choice behavior such as learning rate, reward/loss outcome sensitivity and choice stochasticity.

Earlier meta-analyses of learning, decision-making and reward processing in major depression have not found substantial alterations in learning rate, but have suggested more pronounced impairments in reward and/or loss sensitivity (or equivalently, choice stochasticity) (Halahakoon et al., 2020; Huys, Pizzagalli, Bogdan, & Dayan, 2013). This may be related to anhedonia, which is one of the core symptoms of MDD (American Psychiatric Association, 2013), and which has been linked with a reduced sensitivity to the magnitude of reward or loss outcomes. In contrast to the work suggesting that depression is associated with impaired outcome sensitivity, a recently completed meta-analysis using a novel analytic approach, suggested that patients might be more likely to modify their behaviors in the face of punishments, indicating a higher learning rate in this domain (Pike & Robinson, 2022). A second recent study also found evidence of learning rate changes in depression, although in this case reduced reward learning rate (somewhat surprisingly, increased reward sensitivity was also found), and indicated that the learning rate impairment could be ameliorated by cognitive behavioral therapy (Brown et al., 2021). The literature is therefore somewhat inconsistent with regard both to whether depression is associated with changes in learning rate or outcome sensitivity and whether this change is predominantly seen for positive relative to negative outcomes.

Of note, the majority of RL studies in depressed patients to date has assessed learning from binary outcomes (e.g. outcomes of magnitude 1 or 0) and has probed learning from positive and negative outcomes in separate tasks or trials. The parameters recovered from RL models can depend heavily on the specific task used to estimate them (Eckstein et al., 2022). In other words, finding a reduced outcome sensitivity or learning rate in depressed patients with one type of task does not mean that patients will show this behavior in other tasks. This leaves open the question of whether and how depression is associated with changes in learning from tasks with continuous outcomes and in situations where choices result in both positive and negative sequelae. Answering these questions may go some way to clarifying the inconsistent literature on RL abnormalities in depression.

**Figure 1.**
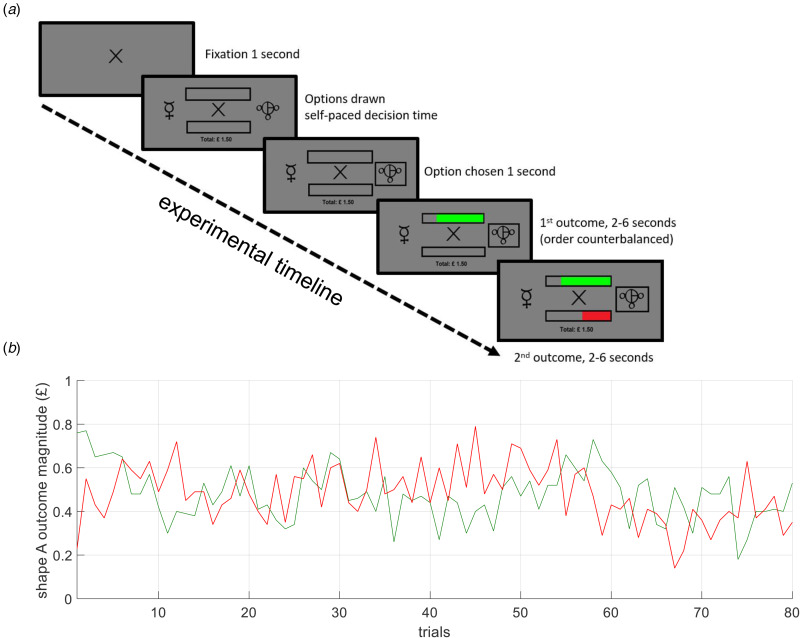
The magnitude learning task. (*a*) On each trial participants were presented with two abstract shapes and were asked to choose one of them. The empty bars above and below the fixation cross displayed the win and loss outcomes, represented by bar fillings in green and red colors, respectively. Here, the full length of the bars was equivalent to £1 for both wins and losses, and the length of the bar fillings from the side of the chosen shape, represented the outcome magnitudes (e.g. if 80% of the upper bar is filled with green, this would mean 80p win). Most importantly, the empty portions of the bars after the green and red fillings indicated the win and loss outcome magnitudes in the unchosen option, respectively; allowing participants to infer which shape would have been the better option on any given trial. (*b*) The outcomes presented to participants during the task. The outcome schedule was designed such that win and loss outcomes were decorrelated (r(79) = −0.032, *p* = 0.78) to require differential learning from wins and losses. The y-axis represents the magnitude of wins (in solid green) and losses (in solid red) associated with shape ‘A’ (in pence units). In total, the task consisted of 80 trials.

In the current study, we assessed the association of learning and decision-making parameters with MDD diagnostic status in a task that required behavioral adjustment to variable magnitudes of both reward and loss outcomes (Fig. 1). In order to assess the degree to which our findings were indicative of trait v. state associations, we compared control participants to two separate clinical groups: patients with current MDD (i.e. who have high state and trait depression) and patients with remitted MDD (i.e. who have high trait and low state depression). Given the somewhat more consistent previous evidence that depression is associated with reduced outcome sensitivity/increased choice stochasticity rather than altered learning rates (Halahakoon et al., 2020; Pike & Robinson, 2022), we predicted that patients with current depression would display comparable learning rates but impaired outcome sensitivity parameters relative to control participants. We did not have a strong a priori hypothesis about whether this would be a specifically state effect (i.e. seen only in currently depressed patients) or whether it would be a trait effect (i.e. seen in both currently and previously depressed patients).

## Methods

Participants were recruited from the local community via posters and social media advertisements. The study involved multiple visits to the research center and all participants attended a screening session in their 1st visit, during which the Structured Clinical Interview for DSM-V (mood disorders and anxiety disorders sections) was administered by a consultant psychiatrist (MB). This clinical assessment was used to confirm eligibility of the participants for one of the three study groups: (1) current MDD group, who satisfied the criteria for a current episode of MDD, (2) patients who had at least two previous episodes of depression lasting longer than 2 weeks but were currently remitted without medication (rMDD) and (3) people who had never met criteria for a mood, anxiety, substance misuse or somatoform disorder. Participants were excluded if: (i) they were receiving any treatment for depression (including medication or any form of psychotherapy), (ii) they met criteria for bipolar disorder, (iii) they had used any street drugs within the last 3 months. Participants in groups 1 and 2 were included if they had co-morbid anxiety disorders, participants in group 3 had no anxiety disorders. Participant groups were matched for gender, age, and years of education (see Table 1) by purposively recruiting patients in groups 2 and 3 to match those in group 1. The study was approved by the University of Oxford Central Research Ethics Committee. Written informed consent was obtained from all participants on the screening visit, in accordance with the Declaration of Helsinki.

**Table 1.** Demographic details of the participants

Variables	Currently depression (*n* = 40)	Remitted depression (*n* = 39)	Healthy control (*n* = 40)	*p*-value
Demographic information:				
Female (%)	80%	79.5%	69.23%	0.474^1^
Age in years, mean (s.d.)	32.93(11.5)	31.73(12.28)	33.3(12.34)	0.854^2^
YoE, mean (s.d.)	17.13(6.09)	18.33(5.85)	18.22(6.16)	0.132^2^
		Questionnaires		
QIDS, mean (s.d.)	13.05(4.62)	4.35(3.48)	2.55(2.33)	<0.001^2^
State-STAI, mean (s.d.)	54.85(9.68)	33.83(8.35)	28.4(7.55)	<0.001^2^
Trait-STAI, mean (s.d.)	62(9.76)	39.7(10.37)	28.98(6.72)	<0.001^2^
SHAPS, mean (s.d.)	31.29(5.09)	21.23(4.50)	21.03(7.09)	<0.001^2^

Note: ^1^p-values were estimated for group differences using a chi-square test. ^2^p-values were estimated for group differences using one-way ANOVA. n, number of participants included in the analysis in this chapter; YoE, Years of Education; QIDS, the Quick Inventory of Depressive Symptoms; State-STAI, state anxiety from the Spielberger State-Trait Anxiety Inventory; Trait-STAI, trait anxiety from the Spielberger State-Trait Anxiety Inventory; SHAPS, the Snaith–Hamilton Pleasure Scale; SD, standard deviation.

The results reported in the current manuscript are based on a behavioral task that was administered in the 1st visit after screening. During the testing session, participants were asked to complete a number of questionnaires, including the Quick Inventory of Depressive Symptoms, 16 items self-report version (QIDS), which measures symptoms of depression (Rush et al., 2003), the Spielberger State-Trait Anxiety Inventory (STAI), which includes separate scales measuring state and trait anxiety (Spielberger, 1983) and the Snaith–Hamilton Pleasure Scale (SHAPS), measuring the ability to experience pleasure (Nakonezny, Carmody, Morris, Kurian, & Trivedi, 2010). They were instructed about the drifting magnitude reinforcement leaning task (see Fig. 1 and legend, and Supplementary Methods for task information). The task was presented on a laptop computer running Presentation software version 18.3 (Neurobehavioural Systems, Berkeley, CA, USA).

In order to dissect individual components of participant choice behavior we fitted different RL models to choice behavior. Similar to our previous work in affective RL (Pulcu & Browning, 2017), we compared three different models: (i) a model with separate learning rates and a single outcome sensitivity parameter, (ii) a model with a single learning rate, but independent reward and loss sensitivity parameters, (iii) a model with independent learning rates and reward/loss sensitivity parameters. Details of the models, a comparison of their ability to account for choice behavior and the generate-recover performance of the best model, which had a single learning rate and independent outcome sensitivity parameters, are provided in the supplementary materials.

**Figure 2.**
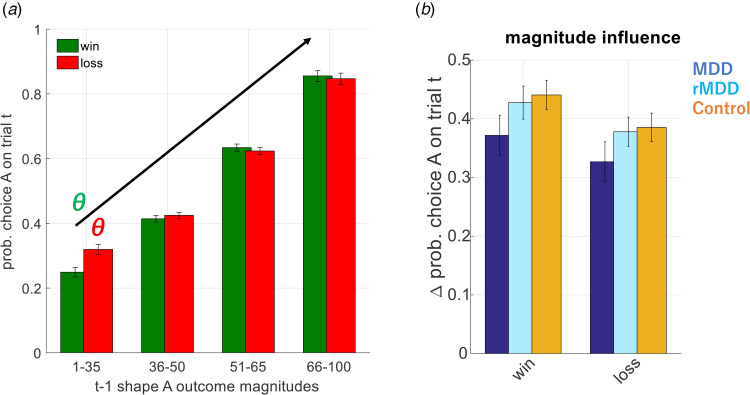
(*a*) Influence of win and loss outcomes on participant choice behavior. A model-free approach demonstrating how outcome magnitudes from trial t-1 influence participant choice probability on trial t. Increasing outcome magnitudes had a greater effect on choice behavior (nb influence of the loss amount plotted as 1-p for ease of viewing). Bars show the mean (SEM) probability of selecting shape A across all participants in the study. A greater effect of outcome magnitude will lead to a steeper slope (*θ*) on this graph. (*b*) The effect of outcome magnitude on choice probability split by group. In this graph, the y axis (delta) is an estimate of slope (*θ*) from panel (A) and is computed as: ((bars 3 + 4)/2-(bars 1 + 2)/2). Bars represent the mean (SEM) of each group, separately for wins and losses. As can be seen the currently depressed group are numerically (but not statistically) less influenced by outcome magnitude than the other groups.

Between group comparisons of demographics and model-free analysis were conducted by using appropriate one-way and repeated measures (rm) ANOVA models with pairwise comparisons to follow-up any statistically significant effects. A simple, non-model based measure of outcome sensitivity in our task is provided by looking at the degree to which participants’ choices are influenced by large *v.* small magnitude outcomes (see Fig. 2 and online Supplementary materials). This measure was used in our model-free analysis. We used three variants of a simple Rescorla–Wagner model coupled with a softmax function to describe participant choice behavior with parameter estimation performed by calculating the joint posterior probability of the parameters. This parameter estimation procedure is identical to our previous work, described elsewhere (Pulcu & Browning, 2017). Group differences in parameters were compared as above using one-way and rm ANOVA models with appropriate follow-up tests.

## Results

The groups were comparable in terms of demographic features (Table 1). As expected, patients with current depression had significantly higher levels of self-reported depression symptom severity, trait and state anxiety and anhedonia scores than the other two groups. The rMDD group had higher scores than the healthy controls on some of the clinical measures (QIDS: t (77) = 2.619; State-STAI: t (77) = 2.844; Trait-STAI: t (77) = 5.463, all *p* < 0.011, Bonferroni corrected). The rMDD group and healthy controls were comparable with respect to SHAPS scores (*p* = 0.882). The clinical features of the current and remitted depression groups are summarized in Tables 2 below.

**Table 2.** Clinical characteristics of current (*N* = 40) and remitted MDD groups (*N* = 39)

Number of previous MDEs	Remitted MDD*	Current MDD**
2	24	14
3	12	6
4⩽	4	20
Life-time axis-I co-morbidity*		
Generalized anxiety disorder	4	11
Panic disorder	20	1
Social anxiety	3	2
Post-traumatic stress disorder	4	6
No life-time co-morbidity	9	20
Seasonal affective disorder	-	3
Dysthymia	-	4
Agoraphobia	-	2
Obsessive compulsive disorder	-	1
Asperger's syndrome	-	1

*In the remitted group all co-morbid disorders were fully remitted at time of study. None of the co-morbid disorders was a likely primary cause of the depressive episodes. ** In the current MDD group, none of the co-morbid disorders was a likely primary cause of the depressive episodes. Patients with MDD were clear of all treatments for at least 6 months prior to their participation in the study.

Analysis of the effect of outcome magnitude on choice behavior (i.e. without fitting of a model; Figure 2), revealed no significant main effect of group (F (2113) = 2.623, *p* = 0.07), and a significant main effect of outcome valence (F (1113) = 5.018, *p* = 0.027). As can be seen from Fig. 2*b*, the MDD group was numerically less influenced by outcome magnitude than the other groups, although this effect was not statistically significant for the comparison with either the control (F(1,76) = 3.194, *p* = 0.078), or the rMDD groups (F(1,77) = 2.902, *p* = 0.092).

Overall performance, measured as the amount of money won in the task, was comparable between the groups (F(2116) = 2.24, *p* = 0.111).

**Figure 3.**
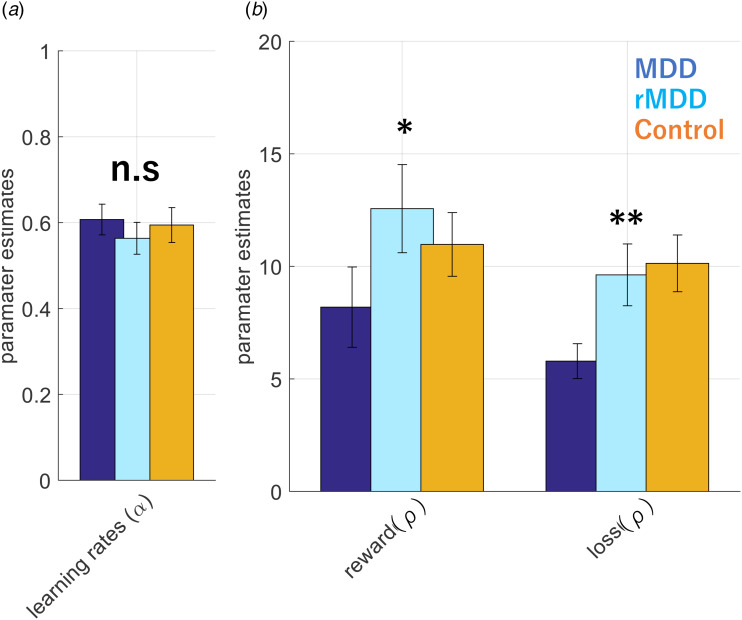
Parameter estimates from the best-fitting reinforcement learning model that explains participant choice behavior. Groups were comparable for learning rates, whereas patients with current depression had significantly lower reward and loss sensitivity parameters. Error bars denote ± 1 SEM. All model parameters are plotted in the normal space for ease of viewing and interpretation.

Based on our previous work modeling participant choice behavior in binary dual-valence RL tasks (Pulcu & Browning, 2017; Pulcu et al., 2019), we utilized behavioral models that can allow valence-specific inferences to be made in learning *v.* reward/loss sensitivity domains (i.e. models with different learning rate and/or outcome sensitivity parameters for win and loss outcomes). The model space included models that relied on (i) a single learning rate, but valence-specific outcome sensitivity parameters; (ii) valence-specific learning rates with a single outcome sensitivity parameter, and (iii) valence-specific learning rates and outcome sensitivity parameters (also see online Supplementary Materials for a detailed description). Model selection between these three models based on Bayesian information criterion scores indicated that the model with a single learning rate (estimated in inverse logistic space) and independent reward and loss sensitivity parameters (estimated in log space) accounted for participant choice behavior better than other competing models (online Supplementary Figure 1). Subsequent 1-WAY ANOVAs conducted on model parameters suggested that there were no significant differences between the groups for learning rate (F (2116) = 0.35, *p* = 0.708, Fig. 3*a*), but that the groups did differ for both reward (F(2116) = 4.35, *p* = 0.0151) and loss sensitivity parameters (F(2116) = 5.51, *p* = 0.005, Bonferroni corrected for two ANOVA models, Fig. 3*b*) relative to rMDD and healthy controls. The MDD group had lower reward and loss sensitivity relative to healthy and rMDD groups (all *p* < 0.031, Fig. 3*b*). These results are independent of the specific model used for analysis, with the same effect being evident when the other models were used. Furthermore, a follow-up sensitivity analysis done by fitting a simple Q-value model [which was insensitive to valence effects by virtue of using a common learning rate and outcome sensitivity parameter] also confirmed that current MDD patients have an overall lower outcome sensitivity term (F(2116) = 4.48, *p* = 0.013).

Finally, we tested whether these effects might additionally be related to symptom severity in the current depression group, such that impairments in reward and loss sensitivity may be more pronounced in patients experiencing symptoms more severely (NB a similar analysis was performed in Brown et al., 2021). However, this effect was not found, with self-reported depression, anhedonia severity or trait anxiety symptoms (e.g. QIDS, SHAPS and trait STAI, Table 1, all *p* > 0.11). The reward and loss sensitivity parameters were comparable within the current MDD group, between patients with (*n* = 13) and without (*n* = 20) comorbid anxiety disorders (both *p* > 0.346).

## Discussion

In this work, we administered a drifting magnitude learning task to groups of patients with current and remitted depression and a group of control participants, in order to probe dynamic learning behavior. We found that patients with current MDD tended to be influenced by the magnitude of negative or positive events to a lesser degree than patients with remitted MDD or healthy controls (Fig. 2). Modeling of participant choices indicated that the behavioral differences observed in the current MDD group were attributable to reduced reward and loss sensitivity rather than to a learning rate effect (Fig. 3*b*).

Using a RL framework, we observed a state dependent effect of depression, that was characterized using a model with two outcome sensitivity parameters and a single learning rate. However, the same group differences were also apparent using a number of other models with different sets of parameters (see online Supplementary information). This suggests that the behavioral signature associated with MDD was independent of the specific assumptions made by each model about whether independent or shared estimates of the learning rate and outcome sensitivity parameters were used for the two outcome valences. This modeling approach allowed us to attribute the behavioral differences observed in the current MDD group to reduced outcome sensitivity rather than altered learning rate. This finding is generally in line with the findings of most meta-analyses of this literature domain (Halahakoon et al., 2020) rather than with the results of a more recent meta-analysis using a novel methodology (Pike & Robinson, 2022), and the result of a recent study by Brown and colleagues (Brown et al., 2021). Of note, in the Brown et al., study, which used a dichotomous outcome of one valence in every trial, there was no difference in reward learning rate between depressed and control participants, but rather there was a correlation between reward learning rate and depression score within the currently depressed group. Using a task in which the magnitude of both reward and loss outcomes varied in every trial we found no similar effect of learning rate either between groups or when correlating with symptom score.

We found a general reduction in outcome sensitivity to both reward and loss outcomes, rather than a specific effect for reward outcomes, or an increased sensitivity to loss outcomes, as has been previously described (Berry, Tanovic, Joormann, & Sanislow, 2019; Hevey, Thomas, Laureano-Schelten, Looney, & Booth, 2017). Clearly, the task used in the current study differs from those described previously, which may account for this difference. The most obvious difference being that, by design, our task necessitates learning from both positive and negative events in parallel, rather than from a single valence of outcome. Secondly, our task utilizes learning from continuous rather than binary outcomes (i.e. learning implicit probabilities associated with reward and/or punishment outcomes). In our experience, continuous outcomes are generally more informative than binary outcomes, resulting in higher average learning rates (Pulcu & Browning, 2017). Our finding, that outcome sensitivity is reduced in this relatively unusual task in depressed patients, indicates that the previously reported reductions of outcome sensitivity in this group are relatively general, rather than being limited to a specific subset of tasks.

The current study describes an association between learning behavior and diagnosis of depression, rather than using an experimental design in which learning behavior is altered. We are therefore unable to comment on the direction of causality between these measures. That is, it is not clear whether reduced outcome sensitivity causes depression, the depression causes reduced outcome sensitivity, or some other factor causes both. Having said this, our finding that outcome sensitivity is reduced in currently, but not previously depressed patients suggests that the association is relevant to state, rather than trait-dependent effects. This suggests that, if a causal effect from outcome sensitivity to depression is assumed, interventions that improve outcome sensitivity would be more likely to reduce symptoms in currently depressed patients rather than prevent symptom development in those at risk.

An important conceptual point when interpreting these results is that the outcome sensitivity parameters in the current paradigm are mathematically identical to inverse temperature parameters, which control decision stochasticity (Browning, Paulus, & Huys, 2022; Huys et al., 2013). This means that the reduced outcome sensitivity seen in currently depressed patients can be equivalently described as increase choice stochasticity (Huys et al., 2013). Thus, while we are able to arbitrate between an effect of learning rate *v.* either outcome sensitivity/choice stochasticity, we cannot definitively attribute the effect to one of these last two processes. We have previously argued that the current literature suggests that depression is associated with reduced outcome sensitivity (Huys & Browning, 2022), but it is important to acknowledge that the current results do not help resolve this specific question. Lastly, currently depressed participants were recruited only if they were not receiving other active treatments. As a result, they are likely to have lower symptom scores than patients who remain symptomatic despite treatment (the average QIDS-SR score in this group was in the moderately depressed range). It therefore remains possible that more severely depressed individuals might show more extensive RL changes than reported here.

In the current study we tested how depressed patients learn about and choose between options of different valence with changeable magnitude. Our results suggest that currently, but not previously, depressed patients are relatively insensitive to the magnitude of both type of outcome. This is consistent with a general reduction in outcome sensitivity, rather than learning rate, being the characteristic impairment of RL in depression.

## Supporting information

Pulcu et al. supplementary material Pulcu et al. supplementary material

## Supplementary material

The supplementary material for this article can be found at https://doi.org/10.1017/S0033291723002520.
