# Supplementary material for: Depression is associated with reduced outcome sensitivity in a dual valence, magnitude learning task
Source: Psychol Med. 2023 Sep 14;54(3):631–6. doi: 10.1017/S0033291723002520 (PMC11443165; doi:10.1017/S0033291723002520)
Supplement: Pulcu et al. supplementary material [file S0033291723002520sup001.pdf]

Supplementary Methods

Drifting magnitude learning task. The drifting magnitude learning task (Figure 1) was adapted from a structurally similar learning task that we previously reported in an earlier publication (Pulcu & Browning, 2017). Participants were presented with two abstract fractals (selected from the Agathodaimon font) and chose the shape which they believed would result in the best outcome. On each trial, the win and loss outcomes were independently generated (both had variable magnitudes between 1 to 99 pence) such that each option would have a win and a loss amount on every trial. As the two outcomes were independent participants had to learn them separately. We minimised the correlation between win and loss outcome sequences to encourage parallel learning from the outcomes associated with the chosen shape ($r(78)=-.032$, $p=.782$). The win and loss outcome sequences had comparable volatility and trial-by-trial variation characteristics to prompt participants to learn both from these outcomes at a comparable rate. The outcome sequences were designed such that shape A would be the better outcome on roughly 50% of the trials. Keeping the win and loss outcome sequences comparable would allow us to assess valence-specific potential learning and outcome sensitivity differences between the groups in an unbiased way.

In the task, the net amount the participant won was the difference between chosen win and loss magnitudes, which was added to their running total at the end of each trial. For example, if the win magnitude was larger than the loss magnitude the participants would accumulate points, whereas if the loss magnitude was larger than the win magnitude, they would lose points. Participants learned from the outcomes of previous trials what was the most advantageous shape to choose on the current trial. The participants completed a single block of 60 trials. The task was administered as a part of a larger battery of behavioural tasks and the participants accumulated artificial game points which did not translate to actual monetary reimbursement. The task was presented on a laptop computer running Presentation software version 18.3 (Neurobehavioural Systems, Berkeley, CA).

Reinforcement learning models. We modelled participant choice behaviour using Rescorla-Wagner (RW) models linked to a sigmoid function.

The Rescorla-Wagner model posits that learners would update their beliefs about subsequent outcomes based on the prediction error generated on the current trial:

$$m_{(t+1)}= m_{(t)}+ \alpha\, (\Theta - m_{(t)})$$

where *m* is the learner's magnitude estimate for an outcome, Θ is the actual outcome (e.g. win or loss magnitude), α (bound; 0:1) is the learning rate and *t* is the trial number. In models with separate learning rates for win and loss outcomes, parameter α would be implemented as α_{win} and α_{loss} in two RW processes running in parallel in order to capture learning separately and independently from win and loss outcomes.

The outcome sensitivity parameter was embedded in the RW model such that it modulates the subjective magnitude of the outcomes participants observe:

$$m_{(t+1)}= m_{(t)}+ \alpha\, (\rho\, \Theta - m_{(t)})$$

where ρ (bound; 0:∞) designates the outcome sensitivity parameter. In models with two outcome sensitivity parameters (win versus loss) these would be implemented as ρ_{win} and ρ_{loss}

separately in the above equation.

Across all models, participant choices were generated using a sigmoid function in which the action tendency depends on the net difference between the win and loss outcomes on a given trial:

$$Q_A = 1/(1+e^{-(win\text{-}loss)})$$

Where Q_A is the choice probability for choosing shape A on a given trial.

Model fitting procedure. In line with our earlier studies in RL using binary and continuous dual valence learning tasks(Pulcu & Browning, 2017; Pulcu et al., 2019), we implemented a Bayesian model-fitting procedure by which the parameters were estimated from single-subject data by calculating the full joint posterior probability of parameters and computing their means at the single-subject level by integrating the probability of parameters with their corresponding discrete values (i.e. a weighted integration of the parameter distributions). Learning rates were estimated in the inverse logistic space, whereas reward/loss sensitivity parameters were estimated in the log space. Single-subject estimates were then used to compute group-level averages to identify behavioural effects associated with MDD. All statistical analyses on parameter values were done in the space where they were estimated. These values were transformed into the normal space for plotting purposes and ease of interpretation (Figure 3).

Supplementary Figures.

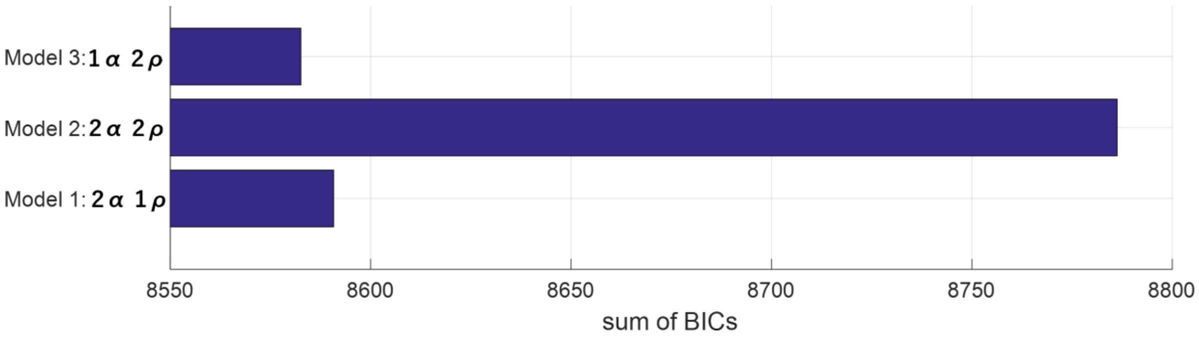

Supplementary Figure 1. Sum of BIC scores demonstrating that the model with a single learning rate and reward and loss sensitivity parameters (i.e., Model 3) explained participant choice behaviour better. Lower bars indicate better model fitting. Summary of a stochastic generate-recover procedure for the best-fitting model is reported in Supplementary Figure 2.

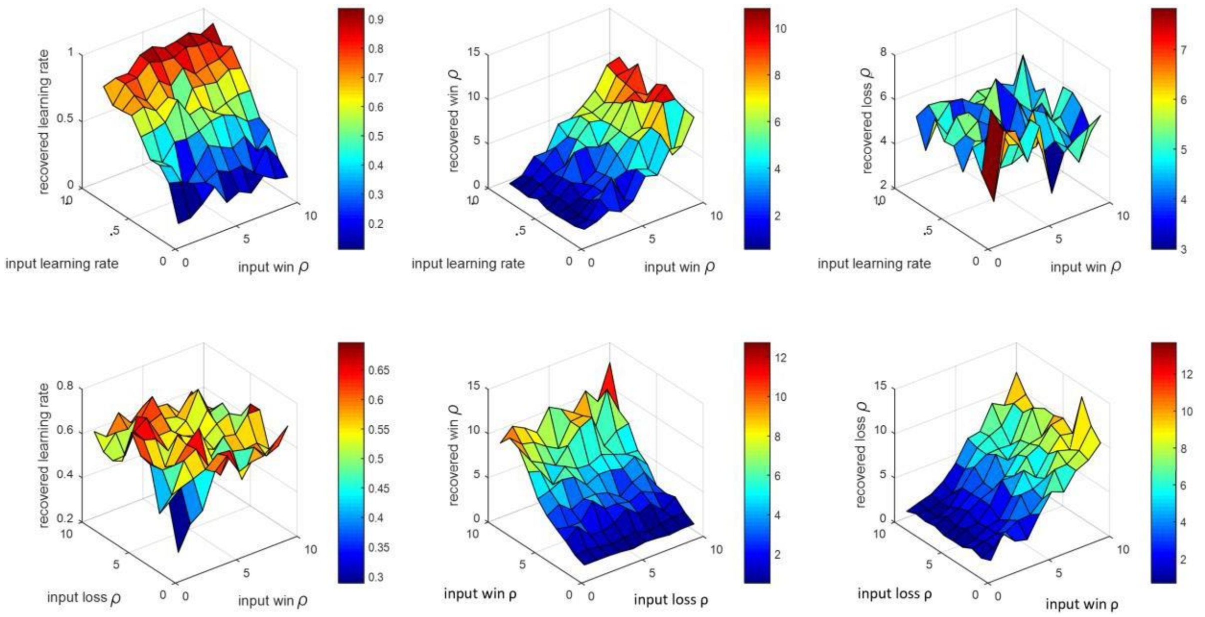

Supplementary Figure 2. Summary of 900 stochastic generate-recover simulations systematically covering all possible parameter values, showing the relationship between the parameters and their estimability on 3D planes. In each panel colour bars designate the value shown by the surface.
